# Supplementary material for: The interactome of intact mitochondria by cross-linking mass spectrometry provides evidence for coexisting respiratory supercomplexes
Source: Mol Cell Proteomics. 2017 Dec 8;17(2):216–32. doi: 10.1074/mcp.RA117.000470 (PMC5795388; doi:10.1074/mcp.RA117.000470)
Supplement: Supplemental Data [file supp_RA117.000470_133922_0_supp_23978_fzffwf.zip › spectra_annotation/mito_spectra_annotation/433-1-4-1-16-1.pdf]

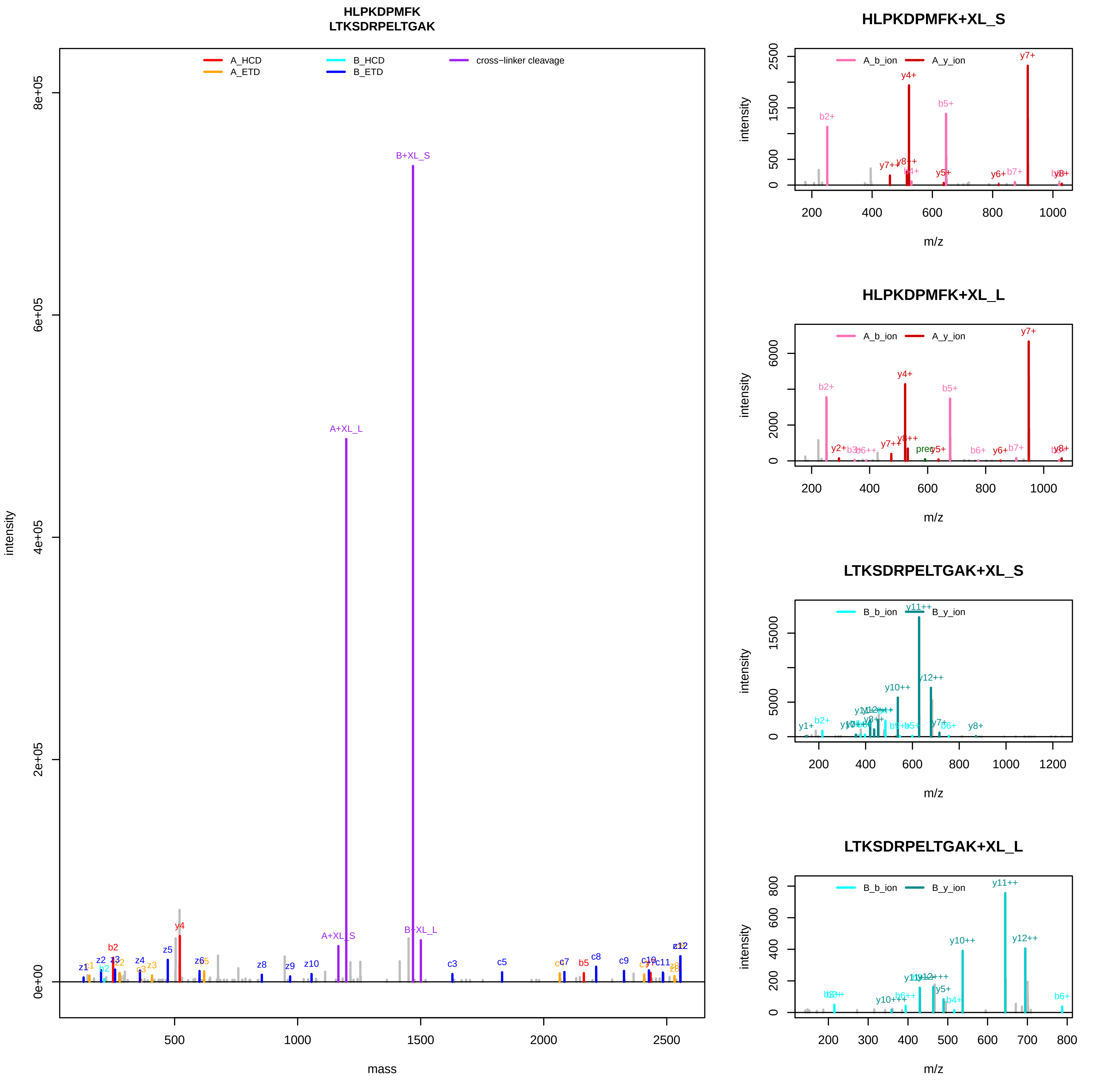

HLPKDPMFK
LTKSDRPELTGAK
A_HCD
A_ETD
B_HCD
B_ETD
cross−linker cleavage
intensity
mass
8e+05
6e+05
4e+05
2e+05
0e+00
500
1000
1500
2000
2500
B+XL_S
A+XL_L
A+XL_S
B+XL_L
y4
b2
b5
z1
z2
z3
z4
z5
z6
z8
z9
z10
c3
c5
c7
c8
c9
c10
c11
c12
HLPKDPMFK+XL_S
HLPKDPMFK+XL_L
LTKSDRPELTGAK+XL_S
LTKSDRPELTGAK+XL_L
A_b_ion
A_y_ion
B_b_ion
B_y_ion
m/z
y7+
y4+
b5+
b2+
y7++
y8++
y5+
y6+
b7+
y8+
y2+
prec
b6+
y11++
y12++
y10++
y1+
y7+
b6+
y8+
y5+
b4+
